# Comparative metabonomics of Wenxin Keli and Verapamil reveals differential roles of gluconeogenesis and fatty acid β-oxidation in myocardial injury protection

**DOI:** 10.1038/s41598-017-09547-w

**Published:** 2017-08-18

**Authors:** Miaomiao Jiang, Qiuying Wang, Jingrui Chen, Yanan Wang, Guanwei Fan, Yan Zhu

**Affiliations:** 1 Tianjin State Key Laboratory of Modern Chinese Medicine, Tianjin University of Traditional Chinese Medicine, Tianjin, China; 2 First Teaching Hospital of Tianjin University of Traditional Chinese Medicine, Tianjin, China; 3 Institute of Materia Medica, Chinese Academy of Medical Sciences & Peking Union Medical College, Beijing, China

## Abstract

Metabonomics/metabolomics is a rapid technology for comprehensive profiling of small molecule metabolites in cells, tissues, or whole organisms, the application of which has led to understanding pathophysiologic mechanisms of cardiometabolic diseases, defining predictive biomarkers for those diseases, and also assessing the efficacious effects of incident drugs. In this study, proton nuclear magnetic resonance (NMR)-based metabonomics was employed to identify the metabolic changes in rat plasma caused by myocardial ischemia-reperfusion injury (MIRI), and to compare the metabolic regulatory differences between traditional Chinese medicine Wenxin Keli (WXKL) and Western medicine verapamil. The results revealed that energy-substrate metabolism were significantly disturbed by ischemia-reperfusion (I/R) in myocardium and bulk of the key metabolites could be further modulated by verapamil and/or WXKL. Lipid metabolism and amino acid transamination occurred mainly following the treatment of verapamil, whereas glucose oxidation and BCAA degradation were prominently ameliorated by WXKL to content the energy demands of heart. Moreover, both WXKL and verapamil improved the secretions of taurine and ketone bodies to overcome the oxidative stress and the shortage of energy sources induced by ischemia-reperfusion.

## Introduction

Ischemic heart disease has become a worldwide health problem associated with high morbidity and mortality^1^. After myocardial ischemia, thrombolytic therapy or cardiac surgery, myocardial ischemia-reperfusion injury (MIRI) contributes to adverse cardiovascular outcomes as an important factor^2^. It is characterized by an initial restriction of blood supply to heart followed by the restoration of perfusion and concomitant reoxygenation^3^. An imbalance in metabolic supply and demand within the ischemic heart may lead to an exacerbation of tissue hypoxia, damage and dysfunction, which further result in myocardial stunning, arrhythmias, tachycardia, and ventricular fibrillation^4, 5^. Prevention and management of MIRI has become a critical step during the treatment procedures of ischemic heart to avoid its adverse effects.

Currently available Western medicine for preventing MIRI are usually single-targeting molecules, such as an oxygen free radical scavenger, antioxidant, calcium channel blocker, or anti-apoptotic agent^4, 6^. Verapamil is a calcium channel blocker that is utilized clinically to treat hypertension, angina pectoris, and cardiac arrhythmia^7, 8^. It is also used intra-arterially to reduce ischemia reperfusion injury, control arrhythmias, and limit myocardial ischemia size^9^. Traditional Chinese medicine (TCM), especially complex herbal formulations, also has a long history in treatment of ischemic heart diseases in China. Wenxin Keli is a patent Chinese medicine recorded in Pharmacopeia of the People’s Republic of China (the 2010th edition, Z10950026). It is composed of extracts from *Codonopsis pilosula*, *Polygonatum sibiricum*, *Panax notoginseng*, and *Nardostachys jatamansi* as well as amber. A randomized, double-blind, placebo-controlled, parallel-group, and multicenter trial conducted with 1200 eligible participants showed the efficacy and safety of WXKL in patients with frequent premature ventricular contractions^10^. Basic science research confirmed WXKL efficacies on treating atrial fibrillation^11^, ventricular-triggered arrhythmia^12^, myocardial infarction-induced arrhythmia^13^ and Brugada syndrome^14^. Interestingly, in addition to its efficacy on arrhythmia, a number of studies also suggested that WXKL might be effective for treating myocardial infarction in rat^15^ and rabbit^16^ models. Indeed, a recent systematic review and meta-analysis of randomized and controlled trials on WXKL also confirmed a better clinical outcome in the treatment of arrhythmia, angina, and heart failure^17^. Unlike Western medicine with a specific target, TCM, with its characteristic multiple and complex components, is destined to associate with multiple pathological targets and pathways. However, it remains a challenge to depict the exact mechanism of TCM.

Metabonomics is a systems biology approach for understanding the metabolic alterations of living systems in response to pathophysiological stimuli via multivariate statistical analysis of biological proton nuclear magnetic resonance (^1^H-NMR) spectroscopic data^18^. It has been extensively used in diagnosis of diseases, to screen potential biomarkers as well as to discover drug targets involved. In addition, metabonomics may also provide crucial insights into the holistic and synergic effects of TCM^19^. In this study, we employ a rat model of MIRI and utilize metabonomics in combination with molecular biological assays to investigate the associations and differences of metabolic remodeling effects between verapamil and WXKL, with the objective of an in-depth understanding of similar and divergent cardioprotective effects between a Western medicine and a TCM.

## Results

### Echocardiography

**Figure 1:**
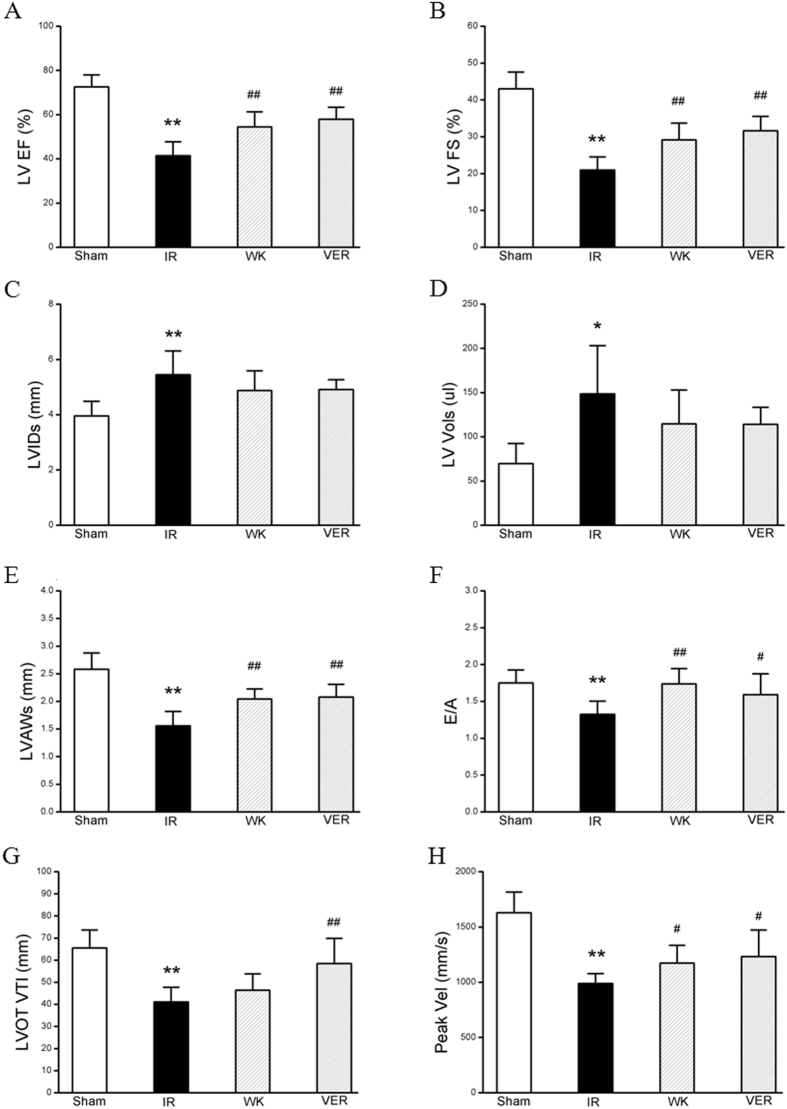
Effects of WXKL and verapamil on rat cardiac function. Quantitative assessment of dilation and systolic function as (**A**) left ventricular ejection fraction (LVEF), (**B**) left ventricular fractional shortening (LVFS), (**C**) left ventricular end-systolic dimension (LVIDs), (**D**) left ventricular systolic volume (LV Vols), (**E**) left ventricular end-systolic anterior wall (LVAWs), (**F**) ratio of E-wave to A-wave (E/A), (**G**) aorta velocity time integral mean velocity (AoV VTI), and (**H**) aortic valve peak velocity (Peak vel). Data are expressed as means ± S.D.; *p < 0.05, **p < 0.01 versus sham group; ^#^ *p* < 0.05, ^##^ *p* < 0.01 versus model group.

The beneficial effect of WXKL pretreatment on cardiac insults induced by ischemia-reperfusion (I/R) was confirmed by quantitative analysis of echocardiography. Results in various groups are summarized in Fig. 1. Compared to Sham group, MIRI group had a significant decrease in left ventricular ejection fraction (LVEF, 72.6 ± 5.40 *vs*. 41.46 ± 6.27, *p* < 0.01), left ventricular fractional shortening (LVFS, 43.03 ± 4.56 *vs*. 20.97 ± 3.56, *p* < 0.01), left ventricular end-systolic anterior wall (LVAWs, 2.58 ± 0.29 *vs*. 1.56 ± 0.26, *p* < 0.01) and ratio of E-wave to A-wave (E/A, 1.75 ± 0.17 *vs*. 1.33 ± 0.18, *p* < 0.01), whereas an increase in left ventricular end-systolic dimension (LVIDs, 3.95 ± 0.53 *vs*. 5.45 ± 0.86, *p* < 0.01) and left ventricular systolic volume (LV Vols, 69.76 ± 22.7 *vs*. 148.72 ± 54.39, *p* < 0.05). All of these were attenuated by WXKL or verapamil after pretreatment for one week. The respective means and standard deviation of WXKL group were: LVEF, 54.43 ± 6.89, *p* < 0.01; LVFS, 29.15 ± 4.55, *p* < 0.01; LVAWs, 2.04 ± 0.18, *p* < 0.01; E/A, 1.74 ± 0.21, *p* < 0.01; LVIDs, 4.88 ± 0.71, *p* > 0.05; LV Vols, and 114.61 ± 38.26, *p* > 0.05. The respective means and standard deviation of verapamil group were: LVEF, 57.93 ± 5.45, *p* < 0.01; LVFS, 31.62 ± 3.92, *p* < 0.01; LVAWs, 2.08 ± 0.23, *p* < 0.01; E/A, 1.59 ± 0.29, *p* < 0.05; LVIDs, 4.91 ± 0.36, *p* > 0.05; LV Vols, and 114.12 ± 19.3, *p* > 0.05. The results of Color Doppler ultrasonography showed that aortic valve peak velocity (Peak vel) and aorta velocity time integral mean velocity (AoV VTI) of MIRI group were significantly lower than those of Sham group (988.23 ± 89.36 *vs*. 1628.20 ± 188.38 and 41.09 ± 6.69 *vs*. 65.48 ± 8.18, respectively, *p* < 0.01). Moreover, these parameters were improved markedly by either treatment of verapamil (1232.79 ± 240.29, *p* < 0.05 and 58.41 ± 11.46, *p* < 0.01, respectively) or treatment of WXKL (1172.95 ± 161.30, *p* < 0.05 and 46.44 ± 7.39, *p* > 0.05, respectively).

### Hemodynamics

**Figure 2:**
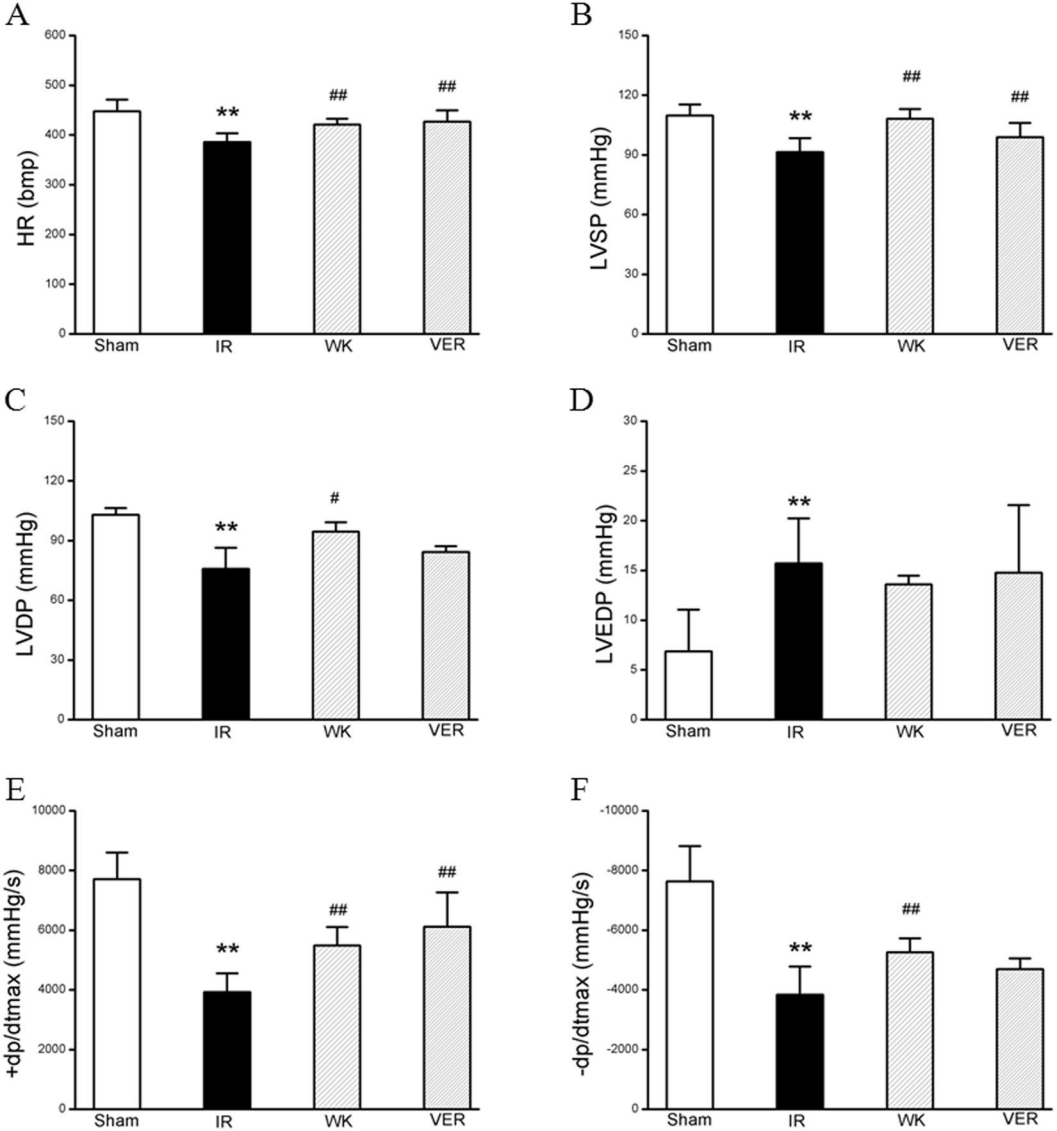
Effects of WXKL and verapamil on hemodynamics post I/R injury. Quantitative hemodynamic assessment was carried out on the left ventricle through right carotid artery to evaluate the role of WXKL or verapamil for I/R injury. (**A**) heart rate (HR), (**B**) left ventricular systolic pressure (LVSP), (**C**) left ventricular development pressure (LVDP), (**D**) left ventricular end diastolic pressure (LVEDP), (**E**) left ventricular maximum upstroke velocity (+*dp*/*dt*_max_), and (**F**) left ventricular maximum descent velocity (−*dp*/*dt*_max_). Data are expressed as means ± S.D.; **p < 0.01 compared with sham group; ^#^ *p* < 0.05, ^##^ *p* < 0.01 compared with model group.

Improvement of the heart functionality by WXKL after I/R injury was further confirmed by hemodynamic measurement in each group. As shown in Fig. 2, in comparison with Sham group, I/R caused a significant decline in heart rate (HR, 447.87 ± 23.42 *vs*. 385.92 ± 17.49, *p* < 0.01) and left ventricular development pressure (LVDP, 103.02 ± 3.44 *vs*. 75.75 ± 10.64, *p* < 0.01), indicating impairments on heart function. Evidently, these impairments were prevented by pretreatment with WXKL (421.37 ± 11.12, *p* < 0.01 and 94.55 ± 4.67, *p* < 0.05, respectively). Meanwhile, WXKL (13.60 ± 0.87) and verapamil (14.76 ± 6.81) had no significant improvement on LVEDP rise induced by I/R injury (15.73 ± 4.52, *p* > 0.05). In MIRI group, the maximum descent velocity (−*dp*/*dt*_max_, −3836 ± 946.21, an evaluation of left ventricular diastolic function), left ventricular systolic pressure (LVSP, 91.48 ± 7.01) and maximum upstroke velocity (+*dp*/*dt*_max_, 3923.50 ± 626.61, an evaluation of left ventricular systolic function), all decreased significantly compared to those of the sham group (−7636.67 ± 1185.33, 109.88 ± 5.54 and 7715.17 ± 887.92, respectively, *p* < 0.01). Significantly, all of these parameters were attenuated by pretreatment with WXKL (−5263.5 ± 462.50, 108.15 ± 4.86 and 5486.50 ± 619.75, respectively, *p* < 0.01).

### Metabolites present in serum samples

**Figure 3:**
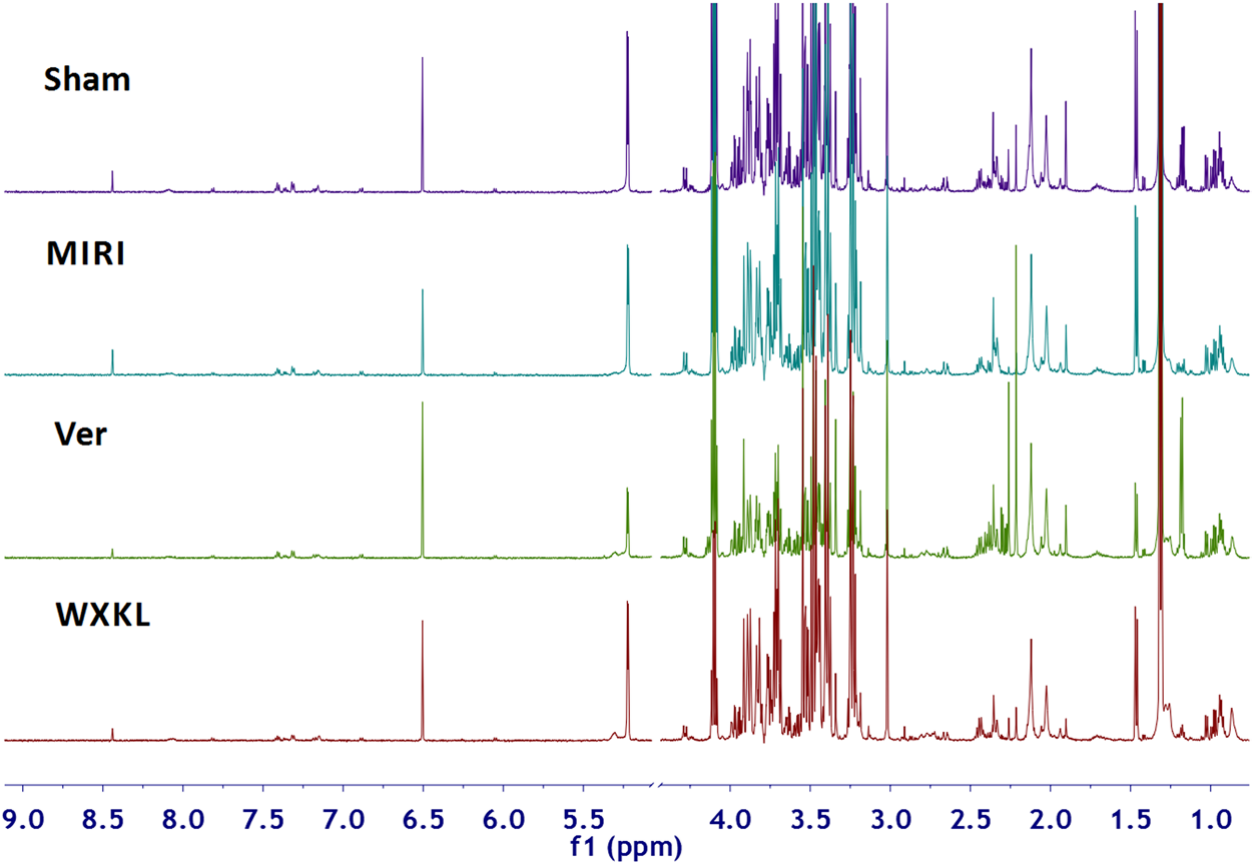
Typical standard ^1^H-NMR spectra of plasma samples collected from sham, MIRI, Ver and WXKL rats. The keys for metabolites are given in Table 1.

Figure 3 shows typical ^1^H-NMR spectra of rat serum samples from the sham, MIRI, Ver and WXKL groups. NMR signals were assigned according to the literatures, standard references and public databases (http://www.hmdb.ca/), which were further confirmed by two-dimensional NMR data. The dominant metabolites present in serum spectra included a range of amino acids such as leucine, isoleucine, valine, alanine, glutamine, phenylalanine, serine, threonine and tyrosine, carboxylic acids such as 3-hydroxybutyrate (HB), lactate, acetate, pyruvate, glutamate, acetoacetate, malate and formate, membrane metabolite choline, lipids, N-acetyl and O-acetyl glycoproteins, acetone, taurine, methanol, glycerol, glycine, and gut microbial-host co-metabolites such as creatine phosphate and trimethylamine N-oxide (TMAO). A detailed NMR assignment is shown in Table 1.

**Table 1:** ^1^H-NMR data for metabolites identified from the rat serum samples.

Peak	Metabolites	*δ* ^1^H (multiplicity^a^)
1	lipids	0.86 (br. s), 1.26 (m), 2.77 (m), 5.28 (m)
2	leucine	0.92 (d), 0.93 (d), 1.70 (m)
3	isoleucine	0.94 (t), 0.99 (d), 3.66 (d)
4	valine	0.97 (d), 1.04 (d), 3.60 (d)
5	3-hydroxybutyrate	1.18 (d), 2.28 (dd), 2.39 (dd), 4.14 (m)
6	lactate	1.31 (d), 4.11 (q)
7	alanine	1.47 (d), 3.76 (m)
8	acetate	1.91 (s)
9	N-acetyl glycoprotein	2.02 (br. s)
10	O-acetyl glycoprotein	2.12 (br. s)
11	acetone	2.21 (s)
12	acetoacetate	2.26 (s)
13	pyruvate	2.35 (s)
14	glutamate	2.12 (m), 2.04 (m), 2.34 (m), 3.76 (m)
15	glutamine	2.13 (m), 2.44 (m), 3.77 (m)
16	malate	2.66 (dd), 2.37 (dd), 4.23 (br. d)
17	creatine phosphate	3.03 (s), 3.93 (s)
18	choline	3.19 (s)
19	trimethylamine N-oxide	3.25 (s)
20	taurine	3.25 (t), 3.40 (t)
21	methanol	3.34 (s)
22	glycine	3.55 (s)
23	threonine	3.58 (d), 1.32 (d), 4.25 (m)
24	glycerol	3.64 (dd), 3.55 (dd), 3.77 (m)
25	serine	3.93 (dd), 3.98 (dd)
26	glucose	5.22 (d), 4.64 (d), 3.89 (dd), 3.83 (m), 3.72 (m), 3.53 (dd), 3.46 (m), 3.40 (t), 3.24 (dd)
27	tyrosine	6.89 (d), 7.18 (d)
28	phenylalanine	7.31 (m), 7.37 (m), 7.41 (m)
29	formate	8.44 (s)

^a^Multiplicity: singlet (s), doublet (d), triplet (t), doublet of doublets (dd), quintet (q), multiplet (m).

### Multivariate data analysis of metabolic changes

**Figure 4:**
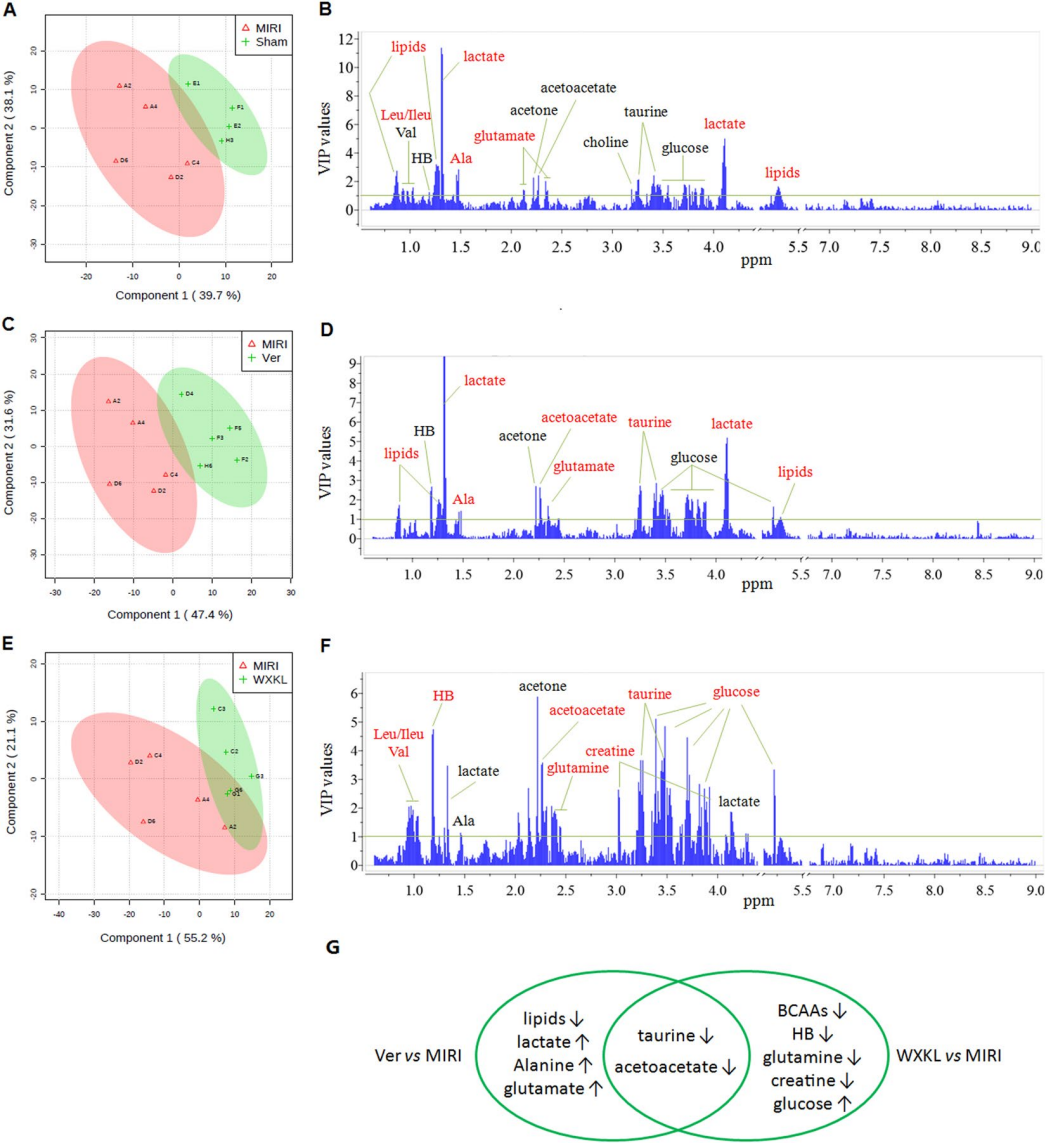
The score plots and VIP plots between (**A**,**B**) sham and MIRI groups, (**C**,**D**) MIRI and Ver groups, (**E**,**F**) MIRI and WXKL groups. (**G**) A summary of the differential metabolites extracted in the three comparison cases.

To explore the metabolic alterations that associated with clustering trends, NMR data of the Sham and MIRI, Ver and MIRI, WXKL and MIRI sets were subjected respectively to supervised PLS-DA analysis and the results of which were exported in both the score plots and VIP plots as shown in Fig. 4. Clear discriminations between Sham and MIRI, Ver and MIRI, WXKL and MIRI groups were observed in the PLS-DA score plots with the first principal component (PC1) explaining 39.7%, 47.4% and 55.2% of the total variances. The corresponding R^2^ and Q^2^ values yielded from cross-validation were 0.68 and 0.32, 0.77 and 0.59, 0.56 and 0.28, respectively, indicating good fit and classification of the models on PC1.

Another fundamental ability of PLS-DA is to select meaningful variables on the basis of VIP values. With a selection criterion of VIP > 1, 13 metabolites were identified for contributing the discrimination between the Sham and MIRI groups, including lipids, three branched-chain amino acids (BCAAs: valine, isoleucine and leucine), three ketone bodies (HB, acetone and acetoacetate), alanine, lactate, glutamate, choline, taurine, and glucose. In the Ver *vs*. MIRI dataset, 9 of the abovementioned metabolites (except for BCAAs and choline) were selected using the same selection criterion. In the WXKL *vs*. MIRI dataset, 10 of the metabolites (except for lipids, glutamate and choline), together with glutamine and creatine phosphate, were extracted for clustering based on their VIP values.

**Figure 5:**
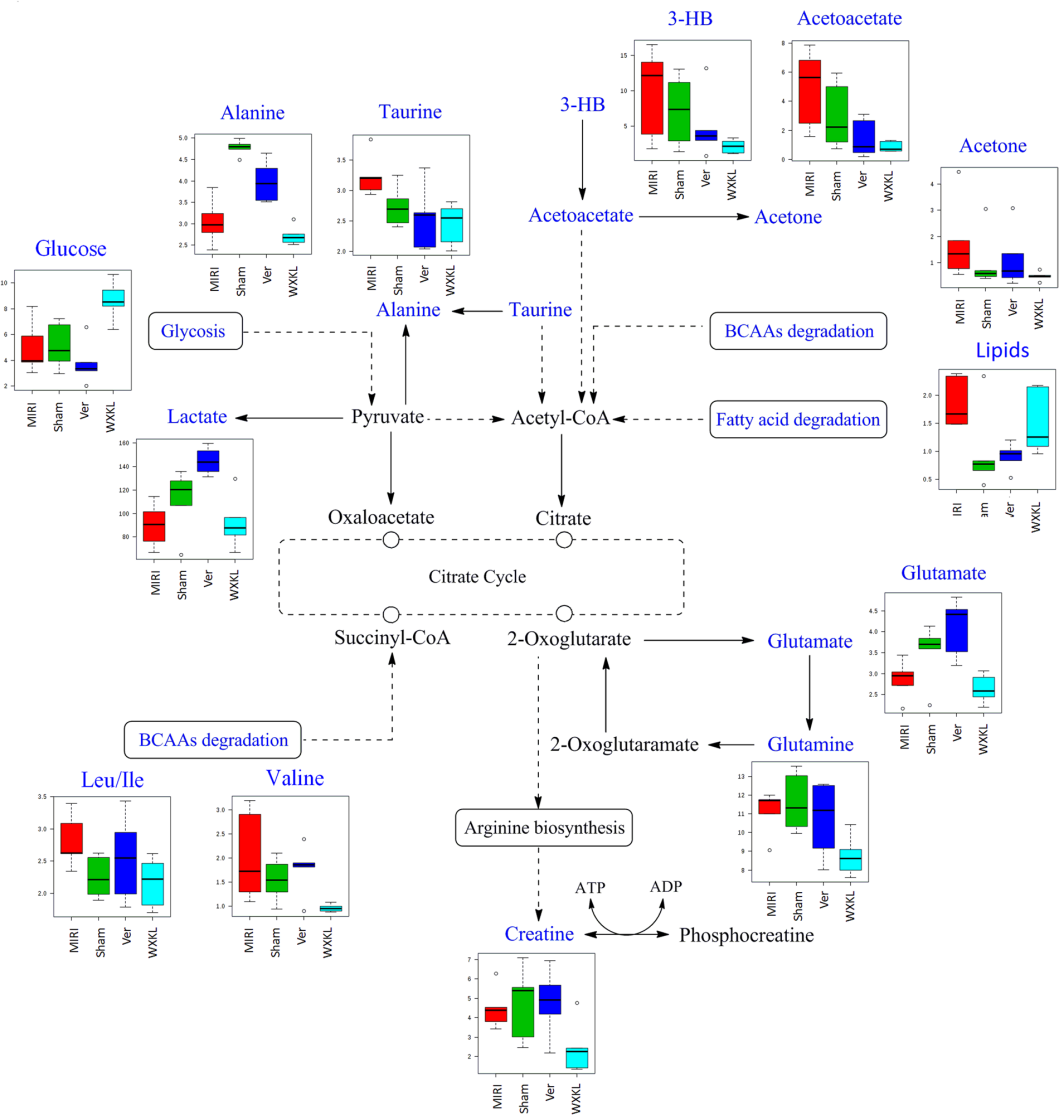
Visualization of the involved pathway for the differential metabolites. The summarized pathway is based on KEGG database (http://www.kegg.jp/).The metabolites in blue were detected in the NMR spectra whereas those in black failed NMR detection. Box plots showed the normalized relative contents of these differential metabolites in the four treatment groups.

Taking the univariate measures (*t*-test) into consideration, I/R injury caused significant elevations of lipids, isoleucine and leucine but reductions of lactate, alanine, and glutamate, compared to those of the control rats with *p* values less than 0.05. Lipids, lactate, alanine and glutamate were uniquely dominant when comparing the Ver to MIRI groups, whereas BCAAs, HB, glutamine, creatine phosphate and glucose were uniquely dominant when comparing the WXKL to MIRI groups. Besides these significantly altered metabolites, two outstanding ones that indicated cluster discrimination were also found in the Ver *vs*. MIRI and WXKL *vs*. MIRI datasets, namely: taurine and acetoacetate. The variation trends of these metabolites and the involved pathway were summarized and presented in Table 2 and Fig. 5.

**Table 2:** The dominating metabolites extracted by multivariate statistical analysis that discriminated the cases of MIRI *vs*. Sham, Ver *vs*. MIRI, and WXKL *vs*. MIRI.

Metabolite (*δ* ^1^H)	PLS-DA (VIP value)	*t*-test (*p* value)	Fold change
**MIRI** ***vs*** *.* **Sham**			
lipids (0.86)	2.38	0.002	2.81
lipids (1.26)	3.21	0.001	6.38
leucine + isoleucine (0.93)	1.52	0.043	1.29
lactate (1.31)	11.39	0.022	0.73
lactate (4.11)	5.02	0.024	0.74
alanine (1.48)	2.86	0.001	0.65
glutamate (2.34)	2.03	0.008	0.75
**Ver** ***vs*** *.* **MIRI**			
lipids (0.86)	1.57	0.003	0.49
lipids (1.26)	2.03	0.005	0.36
lactate (1.31)	12.22	0.001	1.61
lactate (4.11)	5.20	0.001	1.57
alanine (1.48)	1.44	0.021	1.31
acetoacetate (2.26)	2.64	0.036	0.30
glutamate (2.34)	1.69	0.011	1.43
taurine (3.26)	2.47	0.025	0.75
taurine (3.43)	1.18	0.043	0.78
**WXKL** ***vs*** *.* **MIRI**			
leucine + isoleucine (0.93)	1.33	0.036	0.77
valine (1.04)	1.72	0.035	0.47
3-hydroxybutyrate (1.18)	4.57	0.033	0.22
3-hydroxybutyrate (2.28)	1.90	0.020	0.22
acetoacetate (2.26)	3.52	0.012	0.18
glutamine (2.13)	2.70	0.012	0.79
glutamine (2.44)	1.37	0.026	0.65
creatine (3.03)	2.37	0.033	0.55
taurine (3.26)	3.67	0.005	0.64
taurine (3.43)	1.60	0.007	0.75
glucose (5.22)	3.35	0.014	1.74

## Discussion

**Figure 6:**
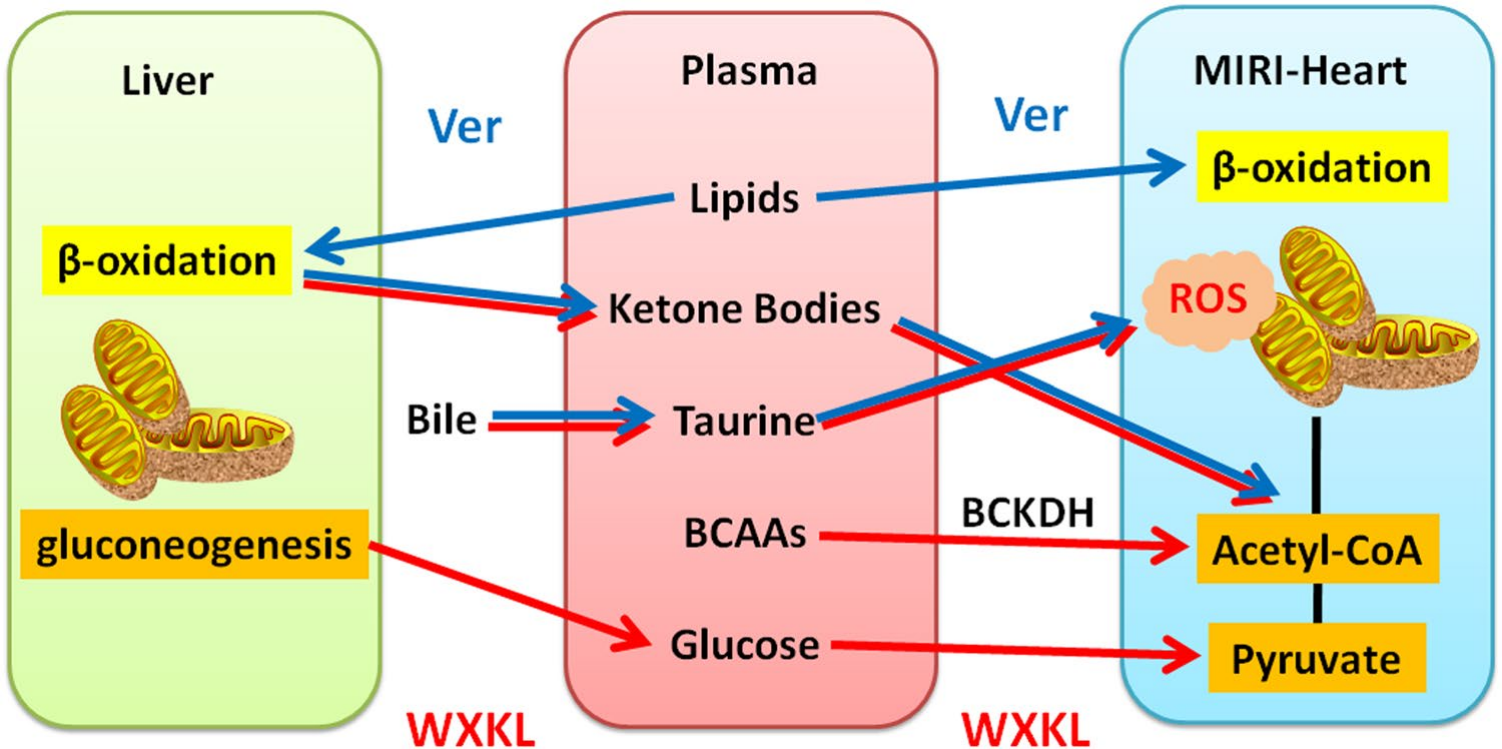
A summary of the metabolic responses of MIRI rats treated by WXKL and verapamil.

Traditional Chinese medicine (TCM), built on a foundation of more than 2,500 years of medical practice, has been widely used in China and is becoming increasingly accepted elsewhere in the world. However, it remains a mounting challenge deciphering the mechanisms of such complex medicine. Metabonomics offers an ideal platform and state-of-the-art approach for the scientific investigation of TCM. It has been successfully used to group TCM symptoms into clinically useful “patterns” for better diagnosis of diseases, and also to provide crucial insights into the holistic assessment of the quality and effectiveness of Chinese medicines^20–22^. In the present study, a systematic metabonomics approach based on ^1^H-NMR technique was performed to compare the metabolic alterations in plasma responding to the modulations of verapamil and WXKL on the myocardial injury induced by I/R. Multivariate statistics analysis for NMR profiles revealed distinct metabolite changes between Sham and MIRI groups mainly associated with the disturbances of myocardial energy substrate metabolism, bulk of which could be further regulated by verapamil and/or WXKL (Fig. 6).

The beneficial effect of WXKL pretreatment on cardiac functions induced by I/R was mainly evaluated by quantitative analysis of echocardiography and hemodynamic assessment. It revealed that an ischemia for 30 min followed by reperfusion for 2 hours in rats led to a decrease in LVEF and LVFS, a reduction of LVAWs and E/A, and an increase in LVIDs and LV Vols. In addition, I/R caused reduction of AV Peak and AoV VTI. All the I/R-induced alterations were evidently ameliorated by pre-administration of WXKL or verapamil for one week, suggesting the potential of WXKL to relieve I/R-induced cardiac insufficiency. After pretreatment for one week, LVDP and −*dp*/*dt*_max_ of the verapamil group were not higher than those of the I/R group. Interestingly, one-week pretreatment by WXKL led to a significant increase in LVDP and −*dp*/*dt*_max_ compared with the I/R group. These results showed that WXKL pretreatment could effectively improve cardiac hemodynamics, increase coronary blood flow, enhance myocardial contractility, and reduce myocardial reperfusion injury in a rat I/R model. Furthermore, WXKL and verapamil are equally protective for heart function by Echo, but WXKL is somehow better by hemodynamics. Previous reports^13, 15, 16^ have also provided additional supporting evidences, including the measurements of infarct area and other crucial biochemical factors, to confirm the effects of WXKL on improving myocardial infarction, ventricular arrhythmias and heart failure.

We hypothesize that the above functional change may be correlated to the metabonomic differences as illustrated in Fig. 6. Circulating lipids and glucose are known to be the predominant energy providing substrates for normal cardiac contractility. Lipids account for the majority (60–80%) of oxidative energy metabolism, while carbohydrates accounts for those remaining 20–40%^23, 24^. After the period of ischemia and following reperfusion, the level of lipids significantly increased, while the level of glucose had no significant change in plasma. In the first stage of ischemia, fatty acids compete with glucose and become the major source of energy with a high rate of uptake resulting in their preferential use in oxidative metabolism^25, 26^. Within minutes of reperfusion, a large burst of ROS is produced^27^. It can induce membrane injury by lipid peroxide formation and damage on fatty acid β-oxidation, further leading to an excessive accumulation of lipids in MIRI plasma. The uptake of glucose from the bloodstream into the cardiomyocytes can be inhibited by high circulating lipid concentrations^28^, hence the level of glucose in MIRI-induced plasma was observed remaining relatively unchanged compared to that of the control group.

Pyruvate, as the last glycolytic intermediate, can further be reduced to lactate, transaminated to alanine, carboxylated to oxaloacetate or malate, or, most importantly, oxidized to acetyl-CoA^29^. Although we failed to detect pyruvate in the present work, lactate and alanine were determined with significantly decreased levels as a result, which indicates a depressed glycolysis in the cardiomyocytes induced by MIRI and leading to limited productions of pyruvate and its derivatives.

Amino acids also can serve as metabolic fuel in muscle and other tissues by the way of transamination. In the reaction, the amino group of an amino acid is transferred to α-ketoacid, typically catalyzed by a transaminase. A very common α-ketoacid is α-ketoglutarate, an intermediate in the citric acid cycle. Transamination of alanine or aspartate together with α-ketoglutarate can give the products of pyruvate or oxaloacetate and glutamate^30^. It is supposed that a decreased level of glutamate caused by MIRI possibly accompanies with a deficiency of pyruvate and oxaloacetate, which contribute as substrates or intermediates in fundamental processes such as glycolysis and the citric acid cycle for further ATP production.

In a worse situation of the MIRI rats, levels of other amino acid sources such as BCAAs (isoleucine and leucine) were elevated in plasma to compensate energy shortages. It suggests that the degradation of BCAAs are probably blocked involving the inhibition of the branched-chain alpha-ketoacid dehydrogenase (BCKDH) complex, leading to a buildup of BCAAs (leucine and isoleucine) and their toxic by-products in the blood. By converting BCAAs either into acetyl-CoA or succinyl-CoA that enter the citric acid cycle, BCKDH complex is a key enzyme for serving BCAAs as energy fuel^31^. The above results reveal that the generations of acetyl-CoA derivatives from complementary energy sources are impaired by IR injury, which further leading to the accumulations of BCAAs in blood, and then failed to afford the greater energy demand.

Compared to the model group, verapamil-treated rats showed a significantly decreased level of lipids, while a fair change of plasma glucose. The results imply that the most beneficial effect of verapamil is related to amendment on mitochondrial function for accelerating the utilization of excessive lipids induced by MIRI. The elevated level of glutamate indicated an amelioration of amino acid transamination to afford relatively sufficient pyruvate and oxaloacetate after verapamil treatment. However, plasma levels of lactate and alanine were observed increasingly in Ver group, which indicate the completeness of glycolytic pathway in cardiac myocytes rather than further formations of acetyl-CoA or oxaloacetate from pyruvate entering the mitochondrion through a transporter.

On the other hand, the WXKL-treated group showed no significant change in the level of plasma lipids while the glucose level was obviously elevated compared with MIRI group. The results imply that WXKL contributes to the supplement of glucose from liver glycogen degradation as energy provision to support the function of the heart, an energy metabolic control distinct from that of verapamil. Previous studies have demonstrated that provision of glucose together with insulin and potassium improves contractile function in the acutely ischemic and reperfused myocardium^32^. Moreover, plasma BCAAs (isoleucine, leucine and valine) showed decreased levels in WXKL-treated rats, suggesting WXKL may also modulate the degradations of BCAAs for providing more Acyl-CoA derivatives to generate ATP.

The metabolic analysis revealed that both verapamil and WXKL could increase the levels of taurine and acetoacetate in plasma compared to those of MIRI group. Taurine is a major constituent of bile with various fundamental biological roles, such as conjugation of bile acids, antioxidation, osmoregulation, membrane stabilization, and modulation of calcium signaling^33^. It is reported essential for cardiovascular function with a specialty of increasing the force and effectiveness of heart-muscle contractions^34^. It also acts as an antioxidant and protects against toxicity of various substances^35^, and the supplementation with taurine has been shown to prevent oxidative stress induced by exercise^36^. Acetoacetate together with 3-hydroxybutyrate are known as ketone bodies that are produced by the liver from fatty acids. During the periods of extreme circumstance, these ketone bodies are readily picked up by the extra-hepatic tissues, and converted into acetyl-CoA which then enters the citric acid cycle and is oxidized in the mitochondria^37^. Pathological states could significantly alter energy metabolism. For example, during the development of heart failure, the capacity of heart to utilize fatty acids is diminished. Recent studies suggested a role of ketone bodies as an alternative fuel and myocardial ketone oxidation as a key metabolic adaptation in the failing human heart^38^. Interestingly, studies of relative oxidation in an isolated heart preparation using *ex vivo* nuclear magnetic resonance combined with targeted quantitative myocardial metabolomic profiling using mass spectrometry revealed that the hypertrophied and failing heart shifts to oxidizing ketone bodies as a fuel source in the context of reduced capacity to oxidize fatty acids^39^. Our results showed that both verapamil and WXKL could stimulate the generations of ketone bodies in liver and transport them through blood to heart for energy. With the elevating levels, verapamil and WXKL are supposed to stimulate bile secreting taurine and liver generating ketone bodies and transport them to heart through blood to resist excessive ROS induced by MIRI, regulate calcium signaling to enhance contractile function, as well as deliver fuels for energy supplements.

In summary, our ^1^H-NMR-based metabonomics approach provides a promising technique to elucidate and compare the cardioprotective mechanisms of traditional Chinese medicine (WXKL) and Western medicine (verapamil) on ischemia-reperfusion injury. Several metabolites, involving some complementary energy sources, and their metabolic pathway were found closely related to the progression of ischemia-reperfusion on myocardium. A prominent mechanism of verapamil was the modulations on lipid metabolism and amino acid transamination in cardiomyocytes, whereas that of WXKL was mainly the regulation of glucose oxidation and BCAAs degradations to content the energy demands of heart. Besides these differences, both WXKL and verapamil can improve the secretions of taurine and ketone bodies to overcome the oxidative stress and the shortage of energy sources induced by ischemia-reperfusion.

Although lipid metabolism fluctuates significantly, it is still a challenge to identify the individual lipid species in a serum sample by ^1^H-NMR spectroscopy without any lipid-enriching step. A lipidomics research based on chromatography coupled with time of fight mass spectrometer (LC-MS) technique would be necessary and is in our subsequent plan to profile more detailed information of disturbance on lipid metabolism by MIRI and its therapeutic efficacy by WXKL.

## Materials and Methods

### Reagents and drugs

Potassium phosphate dibasic trihydrate (K_2_HPO_4_·3H_2_O), sodium phosphate monobasic dehydrate (NaH_2_PO_4_·2H_2_O), disodium fumarate and deuterium oxide (D_2_O, 99.9% in D) were purchased from Sigma-Aldrich (St. Louis., MO, USA). Sodium azide (NaN_3_) in analytical grade was obtained from Tianjin Nankai Share Compounds Co., Ltd. (Tianjin, China). Chloral hydrate was purchased from Tianjin Kermel Chemical Reagent Co. Ltd. (Tianjin, China) and freshly prepared to 5% solution with saline before experiment. WXKL was obtained from Shanxi Buchang Pharmaceutical Co., Ltd (Shanxi, China) and dissolved in saline to a concentration of 0.9 g/mL for experiments. Verapamil was purchased from Tianjin Centralpharm Co., Ltd. (Tianjin, China). We converted a commonly used dosage of verapamil in clinical practice as the chosen dose 20 mg/kg in the experiment.

### Animals

Male Sprague-Dawley (SD) rats, weighing 230–280 g, were purchased from Bio-Technology Co., Ltd., Beijing, Huafukang (Certificate no. SCXK (Jing) 2012-0001). The animals received standard diet and water ad libitum, and were kept under a 12-h light/dark cycle.

### Myocardial I/R model and drug administration

Rats were randomly divided into four groups, including Sham, MIRI, WXKL and Ver groups. Seven days before I/R surgery, the rats in treatment groups were administered with WXKL via gavages in saline at a dose of 9 g/kg and Ver via peritoneal injection at a dose of 20 mg/kg once daily for 7 days. The animals in Sham and MIRI groups received saline at 10 ml/kg at the same time. For MIRI model preparation, rats were subjected to ligation on the proximal left anterior descending coronary artery for 30 min and then released according to the previous studies^40^. Rats were anesthetized with 5% chloral hydrate (300 mg/Kg) by peritoneal injection. Skin preparation was completed and placed in a supine position. After left thoracotomy was performed to expose the heart, the proximal left anterior descending coronary artery (LADCA) was ligated with a 5/0 silk. The suture silk was released after 30 min, allowing reperfusion to occur. Then the thorax was closed, and as soon as spontaneous respiration was sufficient, the rats were released and allowed to recover on an electric blanket. The animals in Sham group underwent the same procedure but without ligation of suture silk.

### Echocardiographic assessment on left ventricular function

The left ventricle function was evaluated 2 h after I/R, using a Vevo 2100 Ultra-high resolution small animal ultrasound imaging system in real time (Visual Sonics Vevo 2100, Canada) with a MS-250 ultrasound scanning transduce. Two-dimensional cine loops and guided M-mode frames were recorded from the parasternal long axis by using 13–24 MHz variable frequency ultrasound scanning transducer. Left ventricular ejection fraction (LVEF), left ventricle fractional shortening (LVFS) and other indicators were measured to determine the left ventricular systolic function. All data were analyzed off-line at the end of the study with software resident on the ultrasound system.

### Hemodynamic evaluation of cardiac function

Two hours after reperfusion, rats were anesthetized with 5% chloral hydrate (peritoneal injection) and the left ventricle was cannulated through right carotid artery, which was connected to a bio-function experiment system MP100-CE (BIOPAC systems Inc., Santa Barbara, California, USA). Briefly, the right common carotid artery was dissected and separated from the connective tissue. A catheter was inserted into the left ventricle through the carotid artery. HR, LVEDP, LVSP, +*d*p/*d*t_max_, and −*d*p/*d*t_max_ were measured at baseline.

Adequacy of anesthesia was controlled by monitoring corneal reflex and the lack of response to toe-pinching. Hearts were excised from rats for further investigation after terminal anesthesia by i.p. injection of 5% chloral hydrate at dose 300 mg/kg and heparin (100U). Euthanasia was performed by excessive inhalation of isoflurane. Death was monitored by the cardiac activity and respiration.

### Sample preparation and NMR measurements

After fasting for 12 h, blood samples were collected by abdominal aortic method. Serum were obtained by the centrifugation of blood at 4 °C, and all the samples were snap frozen in liquid nitrogen and stored at −80 °C until required for analyses. Serum samples were thawed at room temperature and centrifuged at 14 000 rpm for 10 min. A 250-μl supernatant was transferred into 5 mm NMR tubes (Norell, USA) and blended with the same volume of 0.2 M phosphate buffer in D_2_O (pD 7.4) containing 0.1% of NaN_3_.

All NMR spectra were acquired at 298 K on a 600.25 MHz Bruker AVIII HD spectrometer (Bruker BioSpin, Germany) equipped with a 5 mm BBO H&F cryogenic probe. Automatic shimming and adjusting of 90° pulse length were performed for each sample. Standard Carr-Purcell-Meiboom-Gill (CPMG) pulse sequence was used to suppress the residual water signal and also filter the broad signals from the molecules with short T_2_ relaxation times^41^. A total of 64 scans were collected into 32 K data points over a spectral width of 12019.2 Hz with a relaxation delay of 4 s and an acquisition time of 3.07 s. An exponential line-broadening of 0.3 Hz was applied to the free induction decay prior to Fourier transformation. Additional ^1^H, ^1^H-correlation spectroscopy (COSY) and ^1^H, ^13^C-heteronuclear single quantum correlation (HSQC) spectra were recorded on the selected samples for the purpose of resonance assignment.

### Data processing for metabonomic analysis

Acquired ^1^H-NMR data were processed with MestReNova 6.1.1 (Mestrelab Research S. L., Spain). Spectra were carefully phased, baseline corrected and referenced to fumarate (6.504 ppm). The spectral region *δ* 0.6–9.0 was integrated into bins with an equal width of 0.01 ppm. Regions for residual solvent signals of water (*δ* 4.40–5.11) and fumarate (*δ* 5.50–6.76) were discarded during analysis. All remaining spectral bins were regarded as variables and then normalized to the total sum of the integrated area.

Multivariate statistical analysis was conducted using the web server for metabonomic data analysis: MetaboAnalyst 3.0 (www.metaboanalyst.ca). Partial least squares discriminate analysis (PLS-DA) was carried out on the pair to scaled NMR data for group clustering and biomarker identification. The goodness of fit and model validity of PLS-DA were evaluated by the parameters of R^2^ and Q^2^ with leave one-out cross validation. The distinct metabolites as potential markers between classes were weighted through the values of variable importance in the projection (VIP) more than one.

### Statistical analysis

All data were expressed as mean ± S.D. and performed using SPSS17.0 statistical software. One-way analysis of variance (ANOVA) followed by Turkey test for multiple comparisons were used between groups. A value of *p* < 0.05 was considered as statistically significant.

### Study approval

All animal procedures and handlings were approved by the Tianjin University of Traditional Chinese Medicine Animal Research Committee (TCM-LAEC2017003) and in accordance with the National Institute of Health’s Guide for the Care and Use of Laboratory Animals.

## Electronic supplementary material

supplementary information
