# Supplementary material for: Comparative metabonomics of Wenxin Keli and Verapamil reveals differential roles of gluconeogenesis and fatty acid β-oxidation in myocardial injury protection
Source: Sci Rep. 2017 Aug 18;7:8739. doi: 10.1038/s41598-017-09547-w (PMC5562700; doi:10.1038/s41598-017-09547-w)

**Comparative metabonomics of Wenxin Keli and Verapamil reveals differential roles of gluconeogenesis and fatty acid β-oxidation in myocardial injury protection**

Miaomiao Jiang^1^, Qiuying Wang^1^, Jingrui Chen^1^, Yanan Wang^3^, Guanwei Fan^1,2^ & Yan Zhu^1^

^1^Tianjin State Key Laboratory of Modern Chinese Medicine, Tianjin University of Traditional Chinese Medicine, Tianjin, China.^2^First Teaching Hospital of Tianjin University of Traditional Chinese Medicine, Tianjin, China. ^3^Institute of Materia Medica, Chinese Academy of Medical Sciences & Peking Union Medical College, Beijing, China.

Correspondence and requests for materials should be addressed to G. F. ([fgw1005@163.com](mailto:fgw1005@163.com)) or Y. Z. ([yanzhu.harvard@gmail.com](mailto:yanzhu.harvard@gmail.com)).

**Figure S1** The scores plot of PCA on the NMR data of 4 groups.


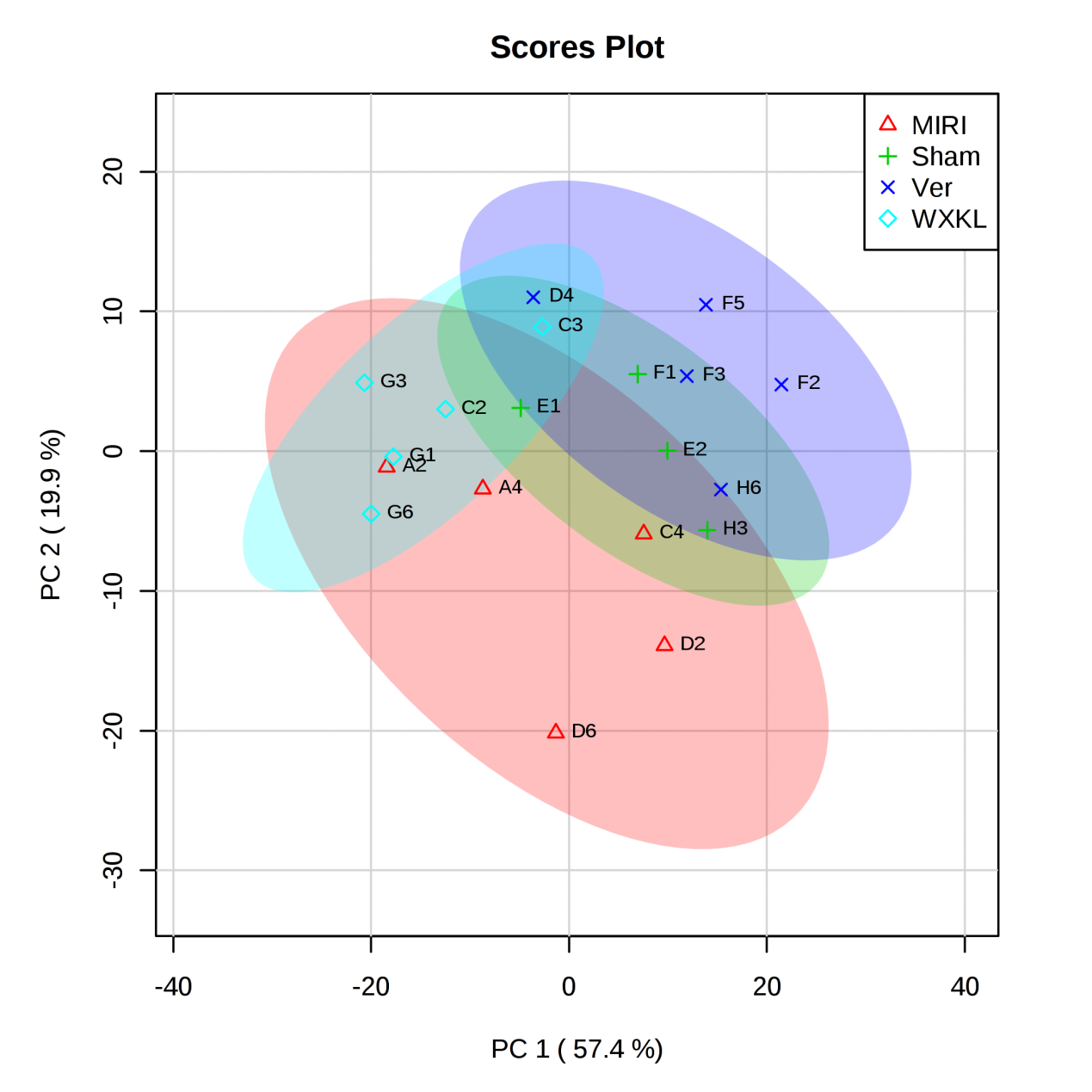


**Figure S2** (A) The permutation plot of PLS-DA between Sham and Model groups; (B) The permutation plot of PLS-DA between Model and Ver groups; (C) The permutation plot of PLS-DA between Model and WXKL groups.


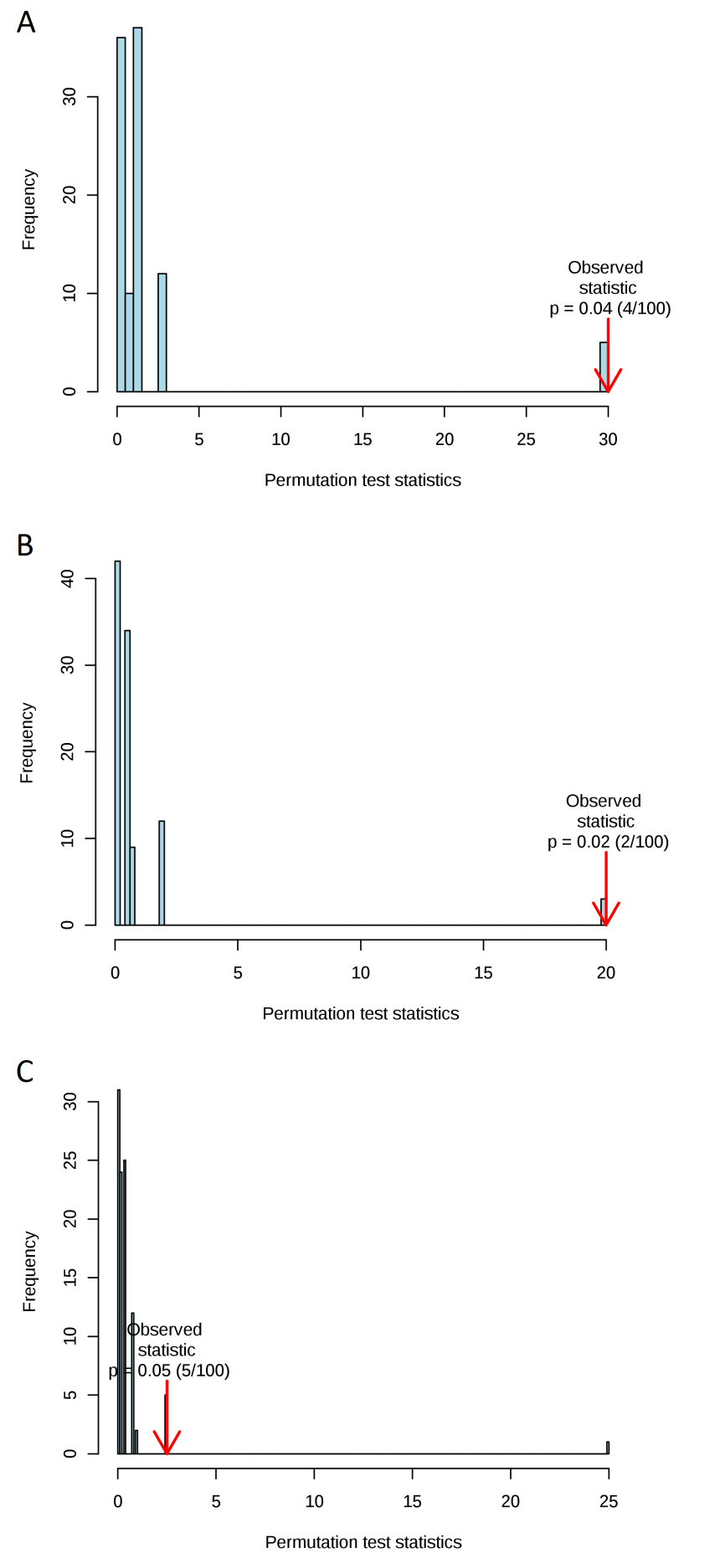

Supplement: Supplementary file 1 — supplementary information [file 41598_2017_9547_MOESM1_ESM.doc]
